# How Can Work Addiction Buffer the Influence of Work Intensification on Workplace Well-Being? The Mediating Role of Job Crafting

**DOI:** 10.3390/ijerph17134658

**Published:** 2020-06-28

**Authors:** Yue Li, Wei Xie, Liang’an Huo

**Affiliations:** 1 School of Public Administration, East China Normal University, Shanghai 200062, China; yli@sem.ecnu.edu.cn; 2 Business School, University of Shanghai for Science and Technology, Shanghai 200093, China; huohuolin@yeah.net

**Keywords:** work intensification, work addiction, job crafting, workplace, well-being

## Abstract

Despite growing attention to the phenomenon of intensified job demand in the workplace, empirical research investigating the underlying behavioral mechanisms that link work intensification to workplace well-being is limited. In particular, a study on whether these behavioral mechanisms are dependent on certain type of individual difference is absent. Using data collected from 356 Chinese health care professionals, this study utilized a dual-path moderated mediation model to investigate the mediating role of job crafting behavior between work intensification and workplace well-being, and the moderating role of work addiction on this indirect path. The results demonstrated that although work intensification was negatively associated with workplace well-being, this effect was more likely to take place for non-workaholics. Specifically, compared with non-workaholics, workaholics were more prone to engage in job crafting behavior in terms of seeking resources and crafting towards strengths, and therefore less likely to have reduced well-being experience. Results are discussed in terms of their implications for research and practice.

## 1. Introduction

One of the consequences arising from accelerated economic, societal, and organizational changes over the past decades is that work has been intensified in numerous occupations [1,2]. Correspondingly, there is a growing attention given by scholars to work intensification which refers to an increase of amount of effort employees need to invest in their work [1]. Work intensification, as a type of job demand, may deplete mental and physical resources of employees and therefore lead to reduced workplace well-being [3,4]. Supporting this notion, previous research has shown that work intensification is related to higher emotional exhaustion and lower job satisfaction above and beyond “conventional” job demands [1,4,5].

According to the transactional theory of stress, it is often the appraisal of job demand rather than job demand itself that triggers individuals’ emotions and cognitions which, in turn, influence their subsequent attitudes and behaviors [6]. Consistent with this, research has shown that positive appraisal of job demand can lead to positive emotions and attitudes [7]. In this sense, positive appraisal of work intensification may also result in less negative responses compared with negative appraisal of work intensification. Indeed, research has proved that, under certain circumstances, work intensification can be viewed in a relatively more positive manner. For example, it was found that a participative climate can make the appraisal of work intensification more favorable, which in turn leads to enhanced employee well-being at the workplace including higher job satisfaction and less emotional exhaustion [2].

Despite this, however, research examining whether certain types of people respond less negatively to work intensification is noticeably absent [8]. One of the individual differences closely related to this issue is workaholism which is also referred to as work addiction [9]. Reflecting one’s tendency to work excessively and being obsessed with work in a compulsive manner [9,10], workaholism is regarded as a form of heavy work investment that can stem from both situational (e.g., organizational climate) and individual (e.g., personality characteristics) factors [11,12,13]. Typical workaholics are motivated by a strong and irresistible internal drive to work hard. They are prone to invest an exceptional amount of time and energy into work even at the expense of other important life roles [14,15]. Brown [8] suggests that, as individuals who place a greater importance on hard work will proactively devote more effort to work, they will be less likely to respond to intensified work in a negative manner. Hence, there is a possibility that work addiction may to some extent buffer the adverse effects of work intensification on individual workplace well-being.

Furthermore, we posit that work addiction could enable employees to cope with work intensification through job crafting. As a type of self-initiated behavior, job crafting refers to employees proactively modifying elements of their tasks to improve the fit between the job and their own skills and preferences [16]. Given that both job demands and resources can represent targets of crafting, job crafting was conceptualized as consisting of seeking resources, seeking challenges, and reducing demands [17]. Considering that workaholics are not particularly eager to reduce their demands and may view work intensification more as a challenge [9,18], we will only turn our attention to the “resources” dimension of job crafting in this study. Apart from seeking resources which refers to behaviors such as asking advice and feedback from colleagues or supervisors [16], we will also focus on another resource-related job crafting behavior named crafting towards strengths.

Recently, there is a growing emphasis on the role of strength use at work as a type of job resource that can lead to desirable outcomes, such as higher level of employee well-being [19] and lower level of burnout [20]. Although organizational supportive practice is a major source for employees to capitalize on their strength, employees themselves can also engage in proactive behavior towards making better use of their own strengths without the involvement of the management [21]. This type of bottom-up employee spontaneous behavior was labeled as crafting towards strengths [22]. Crafting towards strengths can help employees cope with high job demand more effectively [23] and experience higher level of engagement at work [21].

**Figure 1:**
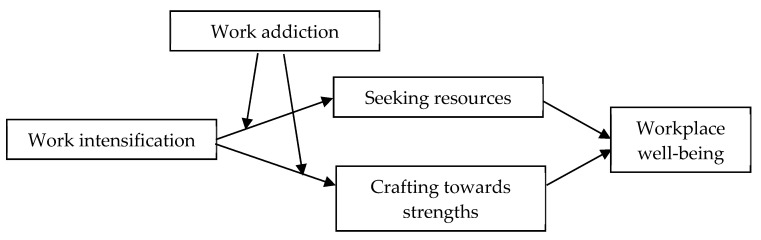
Hypothesized model.

Taken together, we aim to utilize a dual-path moderated mediation model to explore the relationship between work intensification and workplace well-being, the mediating roles of job crafting (including both seeking resources and crafting towards strengths), and the moderating role of work addiction. Figure 1 depicts our proposed model. On one hand, this study can provide us with a better understanding of the behavioral mechanism that links work intensification to employee well-being. While the direct relationship between work intensification and job well-being has been examined by previous research [1,5], the underlying processes accounting for this relationship have received much less attention.

On the other hand, although most research acknowledge that the outcomes of work addiction are negative, such as both poor physical and mental health, there is some debate so far regarding the job well-being related outcomes of work addiction [9]. While some researchers state that a lower enjoyment of work is a defining characteristic of a workaholic who is overly concerned about work [24], some researchers claim that workaholism can lead to the experience of positive affect and satisfaction from work [25,26]. This study aims to provide more insights on this issue and explore the effects of work addiction in the context of work intensification.

### 1.1. Work Intensification and Well-Being

The challenge–hindrance model makes a distinction between challenge and hindrance demands which are differentially associated with well-being consequences [27]. Although challenge demands (e.g., cognitive demands) require effort and energy which may be perceived as stressful, effective dealing with them can foster mastery and competence [28] and lead to job satisfaction, organizational commitment, and work engagement [6,7]. Hindrance demands (e.g., interpersonal conflict), on the contrary, are perceived as potentially constraining personal development and interfering with work achievement [2,29], and thus are likely to induce negative emotion and strain which impair well-being [6,7].

Korunka et al. [5] propose that work intensification should be classified as a type of hindrance demand that leads to impaired well-being. Relying on the job demands-resources model, they further claim that intensified work will not only deplete employees’ resources but also prevent them from getting sufficient opportunity or time for recovery. Combined together, these two processes should result in an increased level of emotional exhaustion [30]. Similarly, some researchers assert that work intensification is a hindrance since it promotes strain and leads to negative work outcomes such as decreased job satisfaction [2]. Work intensification urges employees to work at higher speeds and devote even more effort into their work. Increased pressures from this may lead to work overload. Perceived work overload and time pressure has been linked to negative well-being, including lower job satisfaction, increased job stress, higher work–life imbalance, and more fatigue [31]. Moreover, some research demonstrate that job hindrances are related to lower satisfaction of basic psychological need (need for autonomy, competence, and relatedness), which in turn contributes to poorer well-being, such as lower level of vigor [32,33]. Based on this, we propose the following hypothesis:

**Hypothesis** **1.** *Work intensification is negatively related to workplace well-being.*

### 1.2. Work Intensification and Job Crafting

Although taking anticipatory action for future problems is a key element of proactive behavior [34], job crafting can be responsive and used by individuals as a reaction to deal with the stressful demands placed on them [35]. Research suggests that challenge appraisals of job demand are more likely to trigger the positive emotion and motivation needed for employees to engage in proactive coping behavior, whereas hindrance appraisals tend to prevent employees from actively investing effort in addressing job demands due to the arousal of negative emotions and the feeling of being unable to attain meaningful outcomes [36,37]. Therefore, the relationship between work intensification and job crafting behavior may be moderated by one’s appraisal—only when work intensification is appraised more as challenges rather than hindrances by employees will job crafting be more likely to take place.

Despite that certain types of stressors may be evaluated exclusively as either challenges or hindrances, they can be appraised as both challenges and hindrances simultaneously to different degrees [29]. In line with this, both workload and time pressure have been found to be appraised as a hindrance as well as a challenge at the same time [29,38]. In addition, appraisal of stressor may vary as a function of external environment and individual differences [36]. For example, time pressure is regarded more as a challenge than a hindrance when coupled with job resource such as job autonomy [39].

It has been suggested that workload may be viewed as threatening by employees who perceive a lack of sufficient resources to accomplish the work, while for employees who actively take on more tasks and responsibilities, workload may be regarded as a challenge instead [40]. Individuals scoring high on work addiction are characterized by self-imposed demands and are prone to create more job demand for themselves and take up more challenges and tasks proactively [10,18]. Therefore, they may appraise work intensification in a more favorable manner compared with non-workaholics who may allocate less amount of energy in their work to overcome the work intensification and thus view it more as an obstacle to task accomplishment [40]. In addition, it has been demonstrated that striving for perfection, a characteristic of work addiction [41] is positively associated with challenge appraisal of job demand and active coping behavior [42]. Therefore, it is likely that, compared with non-workaholics, workaholic people may respond to work intensification more as a challenge, which will drive them to engage more in job crafting behavior to overcome the demands imposed by work intensification.

Moreover, given that the process of responding to stressful demands requires a certain amount of energy [43], workaholics, who are prone to devote most of their energy to work, may employ more efforts, such as job crafting behavior, to deal with the demanding situation than their non-workaholic counterparts. Zeijen et al. [15] found that workaholism was positively related to seeking resources. They suggested that this positive relationship could be explained by the lack of social resources associated with workaholism [18] which points towards an obvious need of social resources for workaholics. Further, due to the positive relationship between workaholism and supervisory support [9], workaholism may be particularly related to the “supervisor support” component of seeking resources [15].

In addition, to create a better match between job tasks and personal resources, employees should also focus on their strengths when crafting their jobs [22]. Workaholics are driven by a strong desire to do whatever they feel is important at work [10], they may fully mobilize their resources, including their strengths, to overcome the demands imposed by work intensification [23]. As workaholics are obsessed with their work, they will try to invest all their capabilities in their jobs to attain more work-related goals [10]. Indeed, workaholism has been found to be positively related to increasing structural job resources [15], which include job crafting behaviors such as using one’s capacities to the fullest [44], which is closely related to one’s use of strength at work [20,45]. Based on this, we propose the following:

**Hypothesis** **2.** *Work addiction moderates the relationship between work intensification and seeking resources, such that compared with lower work addiction, higher work addiction leads to more seeking resources in the face of work intensification.*

**Hypothesis** **3.** *Work addiction moderates the relationship between work intensification and crafting towards strengths, such that compared with lower work addiction, higher work addiction leads to more crafting towards strengths in the face of work intensification.*

### 1.3. Moderated Mediation Model

As an important type of job crafting, the positive role of seeking resources in leading to favorable employee outcomes has received support in the existing literature. Job crafting has been regarded as having the potential to help employees create a motivating work environment and improve well-being through increases in job resources such as development opportunities, leader–member exchange, and self-efficacy [46]. Empirically, seeking resources was found to relate positively to work engagement and negatively to exhaustion [47]. A two-wave longitudinal study also showed that increases in job resources (e.g., social support and feedback) positively contributed to work engagement whereas decreases in job resources resulted in more burnout [48]. Employees are likely to experience more work enjoyment on days when they engage in crafting behavior to increase their job resources. This is because having access to expanding pools of resources can, through satisfying the basic needs for autonomy, relatedness, and competence, help employees to enhance intrinsic motivation and to be better able to address job demands [16]. 

Making full use of one’s strength at work is also intrinsically motivating, enjoyable, and energizing in that it can help employees achieve work-related goals [23] and contribute to building psychological capital [49]. Providing an opportunity for employees to engage in tasks that fit their unique strengths is also functional in simulating personal growth and development which can foster an increased identification with work [19]. It was found that strength-related congruence between an employee and the job can foster positive experiences at work such as job satisfaction, pleasure, engagement, and meaning [50]. Moreover, strength use can also help to counteract the strain associated with work stressor and buffer the negative impact of workload and emotional job demands on employee absenteeism [23].

Crafting towards seeking resources and strength use can be regarded as approach (i.e., proactive coping) rather than avoidance behavior because these actions are taken proactively to seek and achieve positive aspects rather than to escape from negative aspects [51]. Proactive coping processes in which individuals accumulate and mobilize resources when they experience stress result from perceiving the situation as challenging [52]. When job characteristics are in alignment with personal needs and skills as a result of job crafting, employees tend to experience more person–job fit [53], which in turn will foster a sense of meaningfulness at work [54]. Following this, it is plausible that compared with non-workaholics, workaholic people tend to display more job crafting behavior towards resources when confronting with work intensification, thereby experiencing more positive well-being. Thus, we propose the following:

**Hypothesis** **4.** *The relationship between work intensification and workplace well-being through seeking resources is conditional on work addiction, such that this indirect path is less negative when work addiction is higher.*

**Hypothesis** **5.** *The relationship between work intensification and workplace well-being through job crafting towards strengths is conditional on work addiction, such that this indirect path is less negative when work addiction is higher.*

## 2. Methods

### 2.1. Sample

Three public tertiary hospitals from Shanghai, the largest seaport city and economic center of China, were selected and contacted with the help of alumni from East China Normal University. Public tertiary hospitals are upper first-class hospitals which provide high-level specialized medical and health services in several regions and conduct higher education and scientific research. They are usually comprehensive or general hospitals at the city, provincial, or national level in China [55]. We presented research-related information to each hospital to voluntarily decide whether or not to participate in the research, and they all agreed. The study was based on a survey of 356 health care professionals employed in the three hospitals. The participants were first contacted and were explained the goals of this research. Next, they were promised that their responses would be anonymous and confidential before they received the research questionnaire to fill out. The questionnaires were translated into Chinese from English using the standard back-translation method prior to their distribution [56]. Furthermore, we interviewed several hospital administrative staff to make sure these translated scales were suitable for Chinese health care professionals. A total of 400 surveys were sent out and 365 completed surveys were returned, yielding a response rate of 86%. After deleting nine forms in which data was missing, we retained a total of 356 effective survey forms. Among the sample, 48% were males; 44.1% were nurses and 55.9% were doctors; 30.6% had a job title at entry-level, 42.1% were at middle level, and the remaining at senior level; the average age was 34 years (*SD* = 8.07 years).

### 2.2. Measures

#### 2.2.1. Work Intensification

Work intensification was measured with 4 items derived from the scale of intensification of job demands [1]. These items attempt to assess the degree to which the amount of effort one needs to put into in daily work increases, such as the need to work with accelerated speed or perform diverse tasks concurrently. A 5-point Likert-type scale was used (1 = strongly disagree to 5 = strongly agree), reliability coefficient α = 93. The sample items include ‘it is increasingly rare to have enough time for work tasks’ and ‘it is increasingly harder to take time for breaks’. 

#### 2.2.2. Work Addiction

Work addiction was measured using 7 items from the Bergen Work Addiction Scale [41] as an indication of the degree to which one feels compelled or an uncontrollable urge to work without relief. A 5-point Likert-type scale was used (1 = strongly disagree to 5 = strongly agree), reliability coefficient α = 0.89. The sample items include ‘Spent much more time working than initially intended’ and ‘Become stressed if you have been prohibited from working’. 

#### 2.2.3. Workplace Well-Being

Workplace well-being was measured using 5 items from Zhang et al. [57]. A 5-point Likert-type scale was used (1 = strongly disagree to 5 = strongly agree), reliability coefficient α = 0.86. The sample items include ‘I find real enjoyment in my work’ and ‘Work is a meaningful experience for me’.

#### 2.2.4. Seeking Resources

This measure was made up of 4 items from the short version scale of job crafting [17], and this scale was also used and validated in the study of Petrou et al. [47]. A 5-point Likert-type scale was used (1 = strongly disagree to 5 = strongly agree), reliability coefficient α = 0.82. The sample items include ‘I ask colleagues for advice’ and ‘I ask my supervisor for advice’.

#### 2.2.5. Crafting Towards Strengths

This measure consisted of 3 items from the scale developed by Kooij et al. [22]. The answering categories ranged from 1 (strongly disagree) to 5 (strongly agree), reliability coefficient α = 0.87. The sample items include ‘In my work tasks I try to take advantage of my strengths as much as possible’ and ‘I look for possibilities to do my tasks in such a way that it matches my strengths’.

#### 2.2.6. Control Variables

Gender, age, profession, and job level were included as control variables to avoid potentially misleading effects on the relationship among variables.

### 2.3. Data Analysis

Correlation and regression analyses were conducted using SPSS Version 23 (IBM, Armonk, NY, USA). Confirmatory factor analysis and moderated mediation analysis were conducted using Mplus Version 6.12 (Muthen & Muthen, Los Angeles, CA, USA).

## 3. Results

### 3.1. Confirmatory Factor Analysis

To examine discriminate validity, we conducted confirmatory factor analysis on the scales including work intensification, work addiction, seeking resources, crafting towards strengths, and workplace well-being (Table 1). Results showed that the fit of the five-factor model in which items were loaded on their respective measures was better than any other model: a one-factor model in which all five study variables were combined; a two-factor model in which work intensification, work addiction, seeking resources, and crafting towards strengths loaded on a single factor, and workplace well-being on one factor; a three-factor model in which work addiction, seeking resources, and crafting towards strengths loaded on a single factor, work intensification and workplace well-being on one factor, respectively; and a four-factor model in which seeking resources and crafting towards strengths loaded on a single factor, work intensification, work addiction, and workplace well-being on one factor, respectively. These confirmatory factor analysis (CFA) results provide support for the validity of the study instrument.

### 3.2. Descriptive Statistics

Means, standard deviations, and correlations of variables in the study are presented in Table 2. As anticipated, work intensification was negatively related to workplace well-being (*r* = −0.20, *p* < 0.01) and positively related to work addiction (*r* = 0.24, *p* < 0.01). Work addiction was positively associated with workplace well-being (*r* = 0.29, *p* < 0.01). In addition, work addiction was positively associated with both seeking resources (*r* = 0.14, *p* < 0.01) and crafting towards strengths (*r* = 0.13, *p* < 0.05). The correlation of seeking resources and crafting towards strengths with workplace well-being were both positively significant (*r* = 0.49 and 0.46, respectively; *p* < 0.01). Variance inflation factors (VIF) were also checked to make sure that this study does not suffer from a problem of multicollinearity. The highest value was 1.789, and there was no VIF exceeding a threshold value of 5 [58]. These results demonstrate that there was no concern for multicollinearity in our study.

### 3.3. Hypothesis Tests

We conducted a hierarchical regression analysis by entering control variables and the study variables into different steps to test the proposed relationship. All the interaction variables were centered in order to attenuate multicollinearity. To test hypothesis 1, we examined the relationship between work intensification and workplace well-being. As shown in Table 3 (Model 1), work intensification was negatively related to workplace well-being (*β* = −0.211, *p* < 0.001) after controlling for all the control variables; therefore, H1 was supported. 

**Figure 2:**
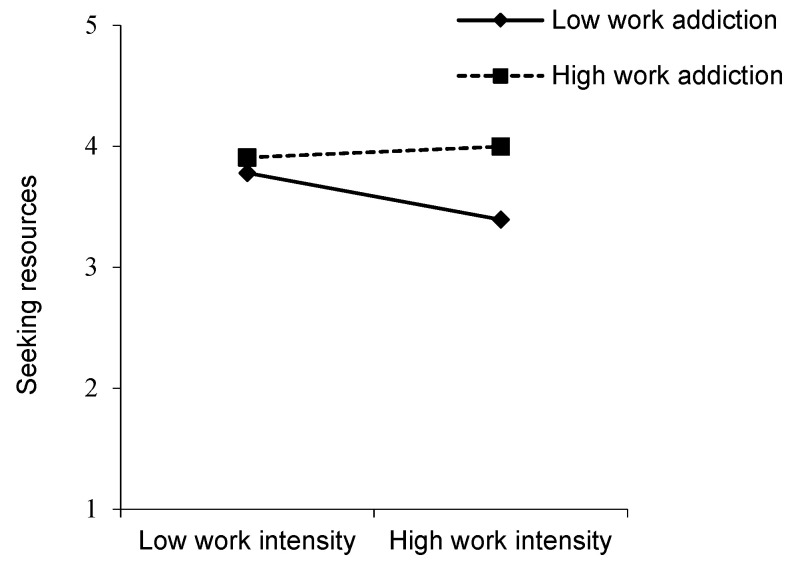
The interaction of work intensification and work addiction on seeking resources.

Hypothesis 2 predicted that the relationship between work intensification and seeking resources would be moderated by work addiction. As shown in Table 3 (Model 5), the interaction term of work intensification and work addiction was positively related to seeking resources (*β* = 0.119, *p* < 0.05). We followed Aiken and West’s [59] procedure for determining the significance of the simple slopes. The simple slope analysis revealed a negative relationship between work intensification and seeking resources for those who were low (1 *SD* below the mean) on work addiction (*β* = −0.119, *p* < 0.05), whereas there was no relationship between these constructs for those who were high (1 *SD* above the mean) on work addiction (*β* = 0.018, *p* > 0.05). The slopes are displayed in Figure 2—when the individual feels a lower level of work addiction, work intensification results in less seeking resources than when the individual feels a higher level of work addiction. Hence, Hypothesis 2 received support.

**Figure 3:**
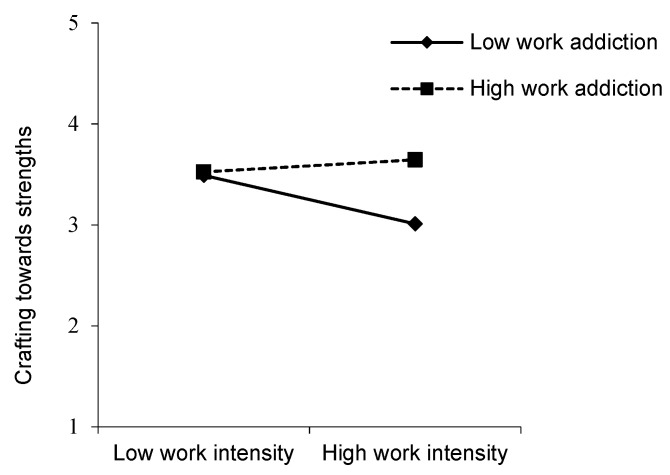
The interaction of work intensification and work addiction on crafting towards strengths.

Hypothesis 3 predicted that the relationship between work intensification and crafting towards strengths would be moderated by work addiction as well. Model 6 of Table 3 reveals that the interaction term of work intensification and work addiction was positively related to seeking resources (*β* = 0.150, *p* < 0.01). We followed Aiken and West’s [59] procedure for determining the significance of the simple slopes. The simple slope analysis revealed a negative relationship between work intensification and seeking resources for those who were low (1 *SD* below the mean) on work addiction (*β* = −0.151, *p* < 0.01), whereas there was no relationship between these constructs for those who were high (1 *SD* above the mean) on work addiction (*β* = 0.025, *p* > 0.05). The slopes are displayed in Figure 3—when the individual feels a lower level of work addiction, work intensification results in less behavior of crafting towards strengths than when the individual feels a higher level of work addiction. Hence, Hypothesis 3 received support.

Next, we tested Hypotheses 4 and 5, where we expected that work addiction would moderate the indirect effect of work intensification on workplace well-being through seeking resources and crafting towards strengths, respectively. Based on the procedure proposed by Preacher and Hayes [60], we first examined the relationship between mediator variables and dependent variable. Model 3 and model 4 in Table 3 reveal that both seeking resources and crafting towards strengths were positively correlated with workplace well-being (*β* = 0.423 and 0.407, respectively; *p* < 0.001) after controlling for the effect of the interaction term of work intensification and work addiction. Then, we adopted the procedure developed by Preacher et al. [61] to further examine the dual-path moderated mediation model with the bootstrapping method. We separated the total effect of work intensification on workplace well-being into direct and indirect effects at different levels of work addiction. The two mediators were entered simultaneously in the same model. The test results are presented in Table 4.

As shown in Table 4, when work addiction was lower (1 *SD* below the mean), the negative relationship between work intensification and workplace well-being through the path of seeking resources was significant (estimate of indirect effect = −0.050, *p* < 0.05, 95% confidence interval (−0.102, −0.013)); whereas when work addiction was higher (1 *SD* above the mean), the indirect effect was not significant (estimate of indirect effect = 0.002, *p* > 0.05, 95% confidence interval (−0.045, 0.053)). Therefore, supporting Hypothesis 4. In addition, when work addiction was lower (1 *SD* below the mean), the negative relationship between work intensification and workplace well-being through the path of crafting towards strengths was significant (estimate of indirect effect = −0.049, *p* < 0.05, 95% confidence interval (−0.101, −0.016)); and when work addiction was higher (1 *SD* above the mean), the indirect effect was not significant (estimate of indirect effect = 0.006, *p* > 0.05, 95% confidence interval (−0.033, 0.054)). Therefore, Hypothesis 5 received support from the data.

## 4. Discussion

Existing research showing the negative impact of work intensification [5,8] indicates that work intensification is a serious problem that must be tackled [2]. To gain more insights into this issue, the current research developed and tested a dual-path moderated mediation model to examine the individual difference that facilitates job crafting behaviors in work contexts characterized by intensified job demand where this self-initiated behavior could lead to higher experience of workplace well-being. The results demonstrated that although work intensification on the whole was negatively related to workplace well-being, this negative impact can be alleviated to some extent by work addiction. In addition, our findings demonstrated that the main effect of work intensification on job crafting was not significant, rather, they were moderated by the level work addiction. Specifically, our study reveals that compared with non-workaholics, workaholics tend to display more job crafting behaviors in the face of work intensification and are thus less likely to experience a lower level of workplace well-being. On one hand, as workaholics are obsessed with work and feel propelled to do whatever they feel is important at work [10], they may have to proactively seek for additional social resources to be able to work extra hard and attain more work-related goals [15], especially in the context of work intensification where one’s resources are more likely to be depleted [4]. On the other hand, workaholics may also try to increase structural resources to make full use of their strengths [15,44], and are thus better able to cope with the demands imposed by work intensification. The finding that people with different level work addiction respond to work intensification in a different manner is consistent with the transactional theory of stress, which indicates that more favorable appraisal of job demand could result in more positive responses from individuals which help them have less negative experiences [6,7].

Although our findings reveal that work addiction could enable employees to cope with work intensification in a more proactive manner to some extent and thereby help them have less negative affective experience, we still must be cautious about interpreting this seemingly “positive” result. The negative effects of workaholism, such as elevated levels of job strain, impaired job performance, and increased work–family conflicts, have been widely acknowledged in the existing literature [11,18]. Even though workaholics may, to some extent, enjoy their work, this experience is more likely to result from an internal compulsion to work rather than intrinsic motivation [9]. As a consequence, the enjoyment workaholics derive from work may be more like an addictive rush rather than true satisfaction with their work [9,62]. Furthermore, the short-term positive relationship between work addiction and work enjoyment may not be enduring. Irresistible obsession with work may prevent workaholics from having sufficient opportunities to recover from their excessive work involvement, thus run the risk of getting emotionally and cognitively drained in the long run [63]. It should be noted that our study demonstrates that even though workaholics may engage in more job crafting in the face of work intensification than non-workaholics, there is no significantly positive relationship between work intensification and their job crafting behaviors. This finding may provide support for the notion that workaholism is in essence an “addiction” to work which, in general, can hardly lead to positive outcomes [62].

Our study also extends on a previous study that focused on the role of cognitive appraisal in linking work intensification to employee outcomes [2], and highlights the importance of proactive behavior in explaining the processes of how work intensification leads to work-related well-being. This finding is in line with existing research that tried to link workaholism directly to coping behavior [14], and makes an extension by showing that workaholism can also act to facilitate one’s proactive coping behavior when encountering an intensified job demand. In doing so, we also add to the existing literature which focused on the facilitating role of employees’ perceived ability and leader’s preferences by incorporating employees’ work style into our framework [37].

In practical terms, the findings of this study demonstrate that interventions should be taken to help health care professionals avoid the negative effects of intensified job demands on their job well-being. Specifically, in order to help an individual better deal with job demands and have less negative experiences in the face of work intensification, job crafting should be supported and facilitated by hospital management, such as investing in a job crafting climate. Hospital supervisors can also educate health care professionals on the virtue of taking crafting behavior, and through coaching, trainings, or empowerment, encourage them to actively increase their job resources and make better use of their strengths.

## 5. Limitations and Future Study

One limitation of this study is that variables were measured from the same source, which could increase the possibility of common method variance (CMV). Both work intensification and workplace well-being reflect individual perceptions, and work addiction reflects individual differences, all of which by their nature require self-reported measures. Moreover, as it might be difficult for supervisors or co-workers to directly observe an employee’s total job crafting behavior, like other research [37], we investigated this behavior with self-rated measures as well. 

On one hand, we tested our hypothesis with a moderated mediation model which is less likely to be detected when relationships are artificially inflated [64]. On the other hand, to check the presence of CMV, we applied Harmon’s single-factor test which revealed that the first factor accounted for only 29.33% of the variance. This result, in combination with confirmatory factor analysis described in the method section, suggests CMV should not be of great concern in our study [65]. Nevertheless, we encourage future studies to collect data at different points in time to investigate our findings. Future research could also use a longitudinal or experimental design to validate the results, as this study was based on a cross-sectional design.

Another point that should be noted here is the use of 1 *SD* above/below the mean as the cut-off value of high/low workaholism in this research for the examination of the moderating role of workaholism. Although this is in line with previous research which examined the moderating effect of workaholism [66], there might be other cut-offs that can be used to capture the degree of high or low work addiction [41]. Results based on Likert ratings may be influenced by the potential effects of cultural orientations on response styles. For example, Japanese employees might score lower on workaholism due to their preference for moderate and less extreme responses and Chinese employees have been found to score higher on workaholism than Western European employees [63]. Due to this, future research could use other standards to classify high and low workaholism to further confirm our results in a Chinese health care context.

In addition, we did not examine directly how workaholics would appraise work intensification which leads to subsequent coping behavior and well-being in this study. Conceptualization of positive/negative features of workaholism may vary across different cultural values, and workaholism may be more positively valued by employees from collectivist societies (vs. individualistic societies) where hard work is valued in and for itself [63], and the appraisal of work intensification may vary accordingly. Moreover, despite the non-significant effect of profession in our study, people with different levels of work addiction may also make appraisals of various degrees in the face of work intensification due to their different profession.

Finally, a prior study found that staff nurses with internal locus of control tend to display less coping behavior as the workload increases, whereas for those who have an external locus of control, a characteristic coupled with workaholism [67], there is no significant relationship between workload and coping effort [43]. Our findings reveal a similar pattern: while workaholics tend to display more job crafting in the face of work intensification than non-workaholics, the relationship between work intensification and their job crafting behavior is not significantly positive. Future studies could explore this further. For example, it may be that different profiles of workaholism exist, such as engaged and disengaged workaholics, which may play a role in determining this relationship [68].

## 6. Conclusions

While most previous research has linked work addiction to negative consequences in the workplace [9], some authors assert that work addiction can lead to positive outcomes [26]. Findings of this study demonstrate that work addiction can drive individuals to display more job crafting behaviors to seek resources and make full use of their strengths when encountering intensified job demand, thus help them have less negative experiences. However, this seemingly “positive” effect of work addiction may just result from a need of workaholics to satisfy their compulsion to work, and the short-term positive effect may not endure in the long run [9,63] as the relationship between higher work addiction and job crafting in our study was actually not that significantly positive.

## Figures and Tables

**Table 1:** Results of confirmatory factor analysis.

	χ^2^	df	RMSEA	CFI	TLI	SRMR
1-factor model	3522.51	230	0.201	0.39	0.33	0.195
2-factor model	3000.16	229	0.184	0.49	0.43	0.189
3-factor model	1885.76	227	0.143	0.69	0.66	0.151
4-factor model	797.35	224	0.085	0.89	0.88	0.063
5-factor model	595.93	220	0.069	0.93	0.92	0.059

Note: RMSEA= Root-Mean-Square Error of Approximation; CFI= Comparative Fit Index; TLI= Tucker-Lewis Index; SRMR= Standard Root Mean-square Residual.

**Table 2:** Descriptive statistics and correlations.

	Mean	SD	1	2	3	4
1. Work intensification	3.64	0.95				
2. Work addiction	2.85	0.86	0.24 **			
3. Seeking resources	3.70	0.65	−0.07	0.14 **		
4. Crafting towards strengths	3.75	0.66	−0.08	0.13 *	0.61 **	
5. Workplace well-being	3.52	0.83	−0.20 **	0.29 **	0.49 **	0.46 **

Note: * *p* < 0.05, ** *p* < 0.01 (two-tailed tests).

**Table 3:** Results of regression analysis.

	Workplace Well-Being								SR		CTS	
	M1		M2		M3		M4		M5		M6	
	SE	β	SE	β	SE	β	SE	β	SE	β	SE	β
Gender	0.088	−0.042	0.082	−0.029	0.073	−0.024	0.074	0.004	0.069	−0.013	0.070	−0.082
Age	0.007	−0.073	0.007	−0.056	0.006	−0.050	0.006	−0.110	0.006	−0.016	0.006	0.133
Profession	0.094	−0.069	0.089	−0.003	0.080	−0.038	0.081	−0.040	0.075	0.083	0.076	0.091
Job level	0.069	−0.009	0.064	−0.030	0.057	0.006	0.058	−0.014	0.054	−0.086	0.055	−0.040
WI	0.046	−0.211 *** (−0.199 ***)	0.045	−0.265 *** (−0.265 ***)	0.040	−0.233 *** (−0.228 ***)	0.040	−0.228 *** (−0.229 ***)	0.038	−0.074 (−0.088)	0.038	−0.090 (−0.094)
WA			0.049	0.354 *** (0.355 ***)	0.044	0.276 *** (0.283 ***)	0.045	0.286 *** (0.292 ***)	0.041	0.183 ** (0.170 **)	0.042	0.167 ** (0.160 **)
WI * WA			0.042	0.118 * (0.120 *)	0.038	0.068 (0.072)	0.039	0.057 (0.065)	0.036	0.119 * (0.114 *)	0.036	0.150 ** (0.141 **)
SR					0.057	0.423 *** (0.425 ***)						
CTS							0.056	0.407 *** (0.393 ***)				
Adjusted R^2^		0.039		0.162		0.333		0.318		0.039		0.051
ΔR^2^		0.052		0.179		0.348		0.333		0.058		0.070
F		3.860 **		10.815 ***		23.118 ***		21.664 ***		3.035 **		3.728 **

Note: WI = work intensification; WA = work addiction; SR = seeking resources; CTS = crafting towards strengths; SE = standard error. Standardized coefficients were reported and those in parenthesis were results of regression model excluding control variables. * *p* < 0.05, ** *p* < 0.01, ***p < 0.001 (two-tailed tests).

**Table 4:** Conditional indirect effect as a function of work addiction.

	Seeking Resources				Crafting towards Strengths			
			95% CI				95% CI	
	IE	SE	LLCI	ULCI	IE	SE	LLCI	ULCI
−1SD	−0.050	0.022	−0.102	−0.013	−0.049	0.021	−0.101	−0.016
+1SD	0.002	0.025	−0.045	0.053	0.006	0.022	−0.033	0.054

Note: CI = confidence interval; IE = indirect effect.
